# Supplementary material for: Seasonal transcriptomic shifts reveal metabolic flexibility of chemosynthetic symbionts in an upwelling region
Source: mSystems. 2025 May 22;10(6):e01686-24. doi: 10.1128/msystems.01686-24 (PMC12172469; doi:10.1128/msystems.01686-24)
Supplement: Supplemental figures — Figures S1 and S2. [file msystems.01686-24-s0001.docx]

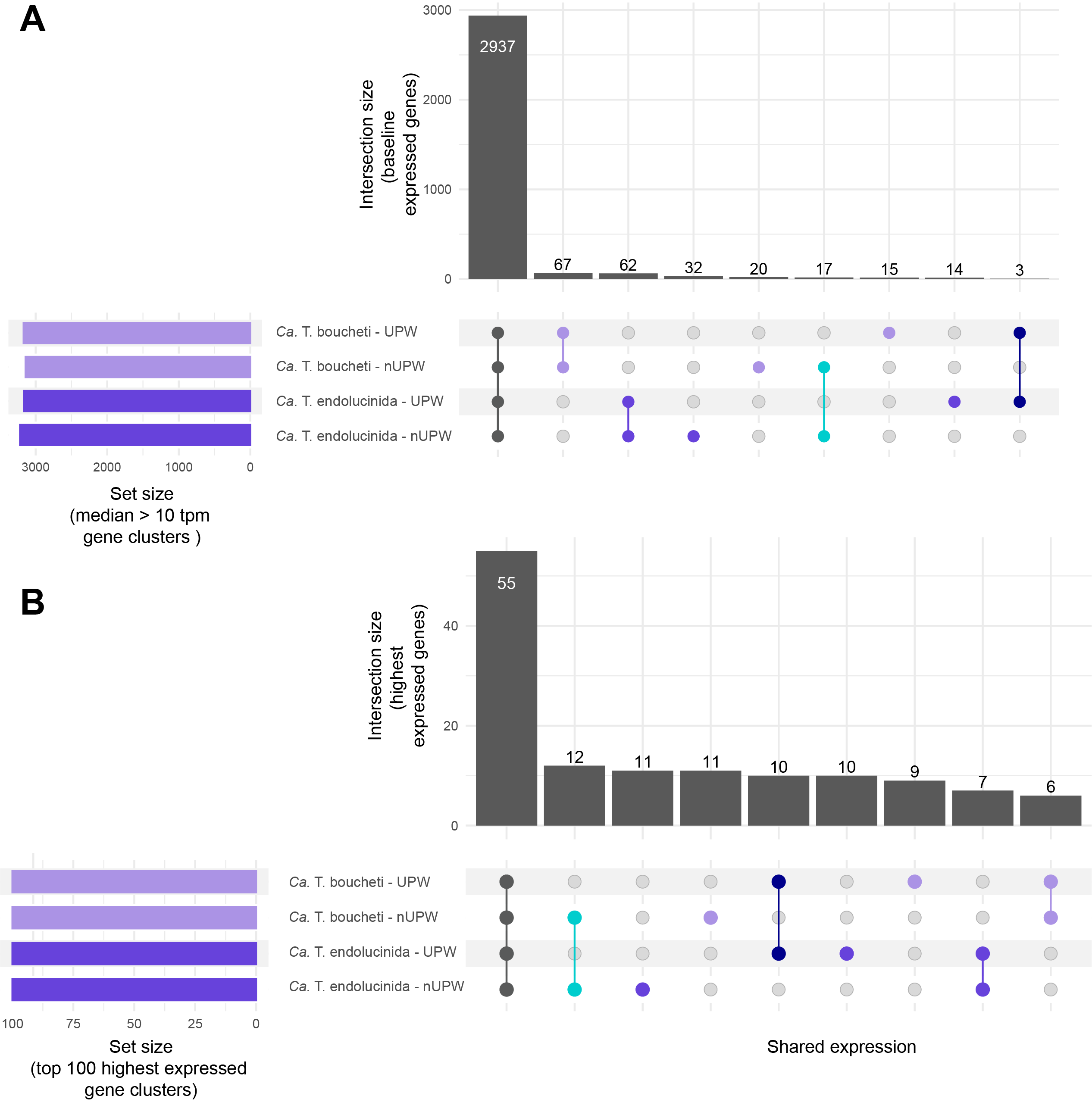


**S1 Figure.** Upset plots representing intersections of transcribed orthogroups. A) Intersections of baseline transcribed orthogroups. B) Intersections of top 100 highest transcribed orthogroups.


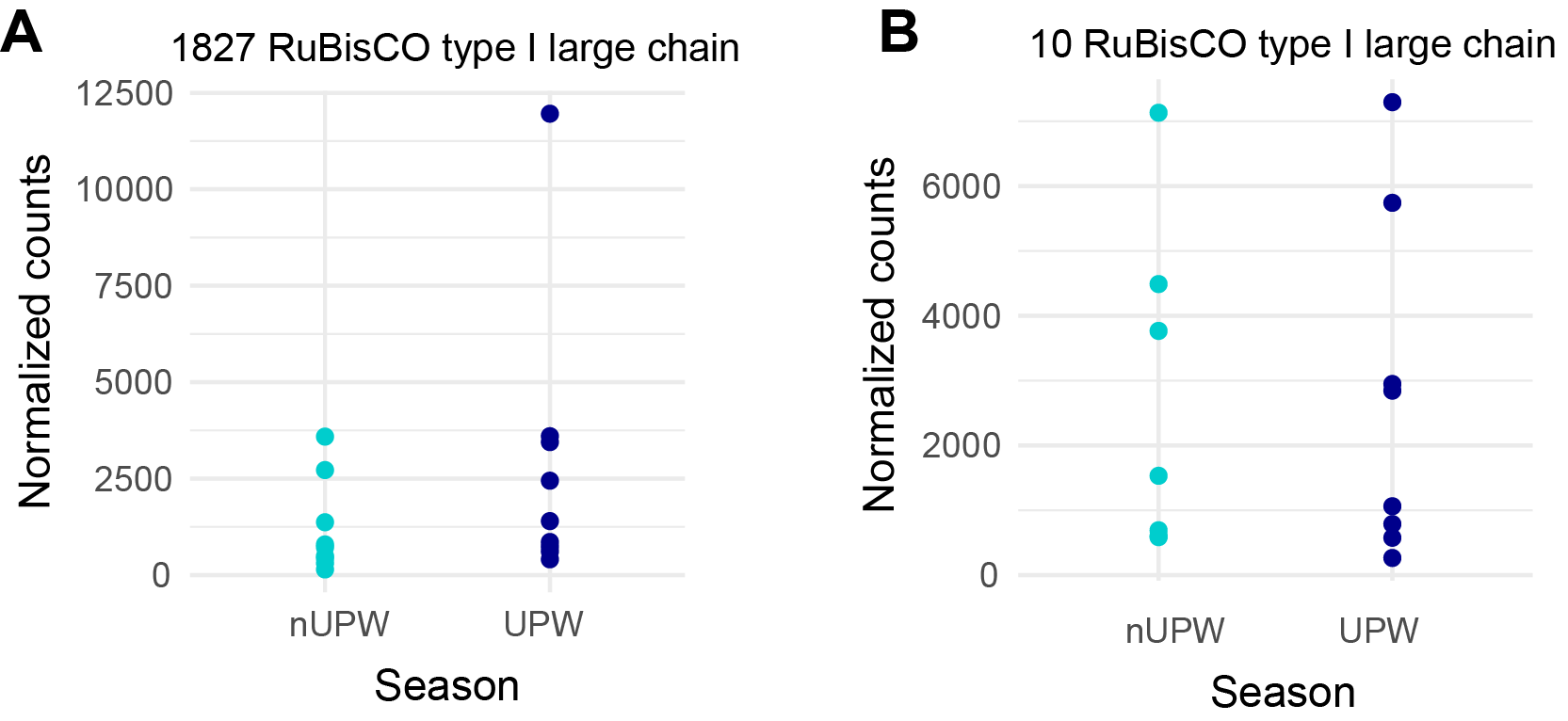


**S2 Figure.** DESeq2 normalized read counts of genes encoding the RuBisCO large chain in A) *Ca.* T. boucheti and B) *Ca.* T. endolucinida TEP
