# Supplementary material for: Seasonal transcriptomic shifts reveal metabolic flexibility of chemosynthetic symbionts in an upwelling region
Source: mSystems. 2025 May 22;10(6):e01686-24. doi: 10.1128/msystems.01686-24 (PMC12172469; doi:10.1128/msystems.01686-24)
Supplement: Supplemental text — Additional methods, results, and discussion. [file msystems.01686-24-s0002.docx]

## Supplementary Methods

### Development of databases for rRNA depletion kit and SortMeRNA

Metagenomic assemblies were generated from the raw reads as described in (Morel-Letelier et al. 2024) to obtain lucinid host and symbiont ribosomal RNA (rRNA) sequences. Barrnap v0.9 [(1)](https://paperpile.com/c/Mmzmxv/bPcY) was used to extract host mitochondrial (*--kingdom mito*), host nuclear rRNAs (*--kingdom euk*), and bacterial (*--kingdom bar*) sequences of the different lucinid species and symbiont clades. The resulting sequences were manually blasted against the NCBI nt database, and hits to molluscan and bacterial sequences were kept. The custom-designed host-specific riboPOOL kit (dp-K024-000089) was designed by siTOOLs Biotech based on rRNA sequences specified in Table I.

**Table I.** Description and NCBI accession of the metagenomes used to extract rRNA sequences and the databases in which the resulting sequences were included

| **Host Species** | **Biosample accession** | **Assembly Sample** | **Geographic origin** | **Depletion kit (host)** | **SortmeRNA (host)** | **SortmeRNA (symbiont)** |
| --- | --- | --- | --- | --- | --- | --- |
| ***Codakia distinguenda*** | SAMN38052585 | coddisantah006 | Guanacaste, Costa Rica | Yes | Yes | No |
| ***Codakia orbicularis*** | SAMN38052626 | codorguadel001 | Guadeloupe | Yes | Yes | No |
| ***Ctena* sp. “COSTE”** | SAMN38052525 | Cteimcahuit010 / Ctena_MEXE | Cahuita, Costa Rica | Yes | Yes | No |
| ***Ctena imbricatula*** | SAMN38052631 | cteimguadel002 | Guadeloupe | Yes | Yes | No |
| **Ctena galapagana** | SAMN38052589 | ctemesantah003 | Guanacaste, Costa Rica | Yes | Yes | No |
| **Phacoides pectinatus** | SAMN38052639 | PhPec3 | Guadeloupe | Yes | Yes | No |
| ***Clathrolucina costata*** | SAMN44767096 | clacoguadel006 | Guadeloupe | No | Yes | No |
| ***Lucinella divaricata*** | SAMN39467987 | DIVDIV /  Lucinella | Elba, Italy | No | Yes | Yes |
| ***Loripes orbiculatus*** | SAMN16952184 | ELBAC10 | Elba, Italy | No | Yes | No |
| ***Ctena decussata*** | SAMN39467948 | L117 | Embiez, France | No | Yes | No |
| ***Loripes orbiculatus*** | SAMN16952203 | LPN | Piran, Eslovenia | No | Yes | No |
| ***Ctena galapagana*** | SAMN38052624 | ctemesantah050 | Guanacaste, Costa Rica | No | No | Yes |
| ***Clathrolucina costata*** | SAMN16952169 | clacocahuit002 | Cahuita, Costa Rica | No | No | Yes |
| ***Loripes orbiculatus*** | SAMN04570537 | LucA | Elba, Italy | No | No | Yes |
| ***Loripes orbiculatus*** | SAMN44767097 | LLP3 | Piran, Eslovenia | No | No | Yes |

### Comparative genomics and transcriptomics

A functional enrichment analysis [(2)](https://paperpile.com/c/Mmzmxv/0Kls6) was performed on the pangenome to identify KOfam functions enriched in each clade. Log fold changes and adjusted p-values were extracted for the corresponding genes (see section Differential gene expression).

## Supplementary results and discussion

### Core symbiotic genes are shared by both symbiont clades and are consistently highly transcribed

**Table II. Core symbiont gene transcription.** Shared orthologues with the top 100 highest transcription levels across symbiont clades and sampling seasons, and their corresponding functional annotations.

| Ortholog ID | Description | Gene symbol | Annotation source |
| --- | --- | --- | --- |
| GC_00000006 | Hypothetical protein | *-* | - |
| GC_00000011 | Cytochrome c | *CYC* | KEGG |
| GC_00000129 | Carbon storage regulator | *csrA* | KEGG |
| GC_00000232 | Cold shock protein | *cspA* | KEGG |
| GC_00000246 | RNA polymerase sigma-32 factor | *rpoH* | KEGG |
| GC_00000460 | Peptidoglycan-binding domain 1 protein | *-* | eggNOG |
| GC_00000472 | Hypothetical protein | *-* | - |
| GC_00000476 | Sulfur-oxidizing protein SoxZ | *soxZ* | KEGG |
| GC_00000489 | 5,6,7,8-tetrahydromethanopterin hydro-lyase [EC:4.2.1.147] | *fae* | KEGG |
| GC_00000514 | Type VI secretion system secreted protein | *hcp* | KEGG |
| GC_00000523 | Gram-negative porin | *-* | eggNOG |
| GC_00000558 | Ribulose-bisphosphate carboxylase large chain [EC:4.1.1.39] | *cbbL* | KEGG |
| GC_00000600 | Sulfur-oxidizing protein SoxY | *soxY* | KEGG |
| GC_00000841 | Malate dehydrogenase [EC:1.1.1.37] | *mdh* | KEGG |
| GC_00000983 | Hypothetical protein | *-* | - |
| GC_00000988 | Hypothetical protein | *-* | - |
| GC_00000989 | Acyl carrier protein | *acpP* | KEGG |
| GC_00001007 | Bacterial DNA-binding protein | *hup* | eggNOG |
| GC_00001014 | Large subunit ribosomal protein L28 | *rpmB* | KEGG |
| GC_00001015 | Belongs to the HesB IscA family | *-* | eggNOG |
| GC_00001041 | Rubrerythrin | *-* | PFAM |
| GC_00001062 | Hypothetical protein |  | - |
| GC_00001096 | Glucose-1-phosphate adenylyltransferase [EC:2.7.7.27] | *glgC* | KEGG |
| GC_00001148 | Hypothetical protein | *-* | - |
| GC_00001203 | Small subunit ribosomal protein S20 | *rpsT* | KEGG |
| GC_00001233 | Hypothetical protein |  | - |
| GC_00001239 | DnaK suppressor protein | *dksA* | KEGG |
| GC_00001334 | Hypothetical protein | *-* | - |
| GC_00001392 | Hypothetical protein | - | - |
| GC_00001479 | DsrC like protein | *-* | eggNOG |
| GC_00001605 | Hemerythrin HHE cation binding domain | *-* | eggNOG |
| GC_00001703 | Modulator of FtsH protease | *yccA* | KEGG |
| GC_00001723 | Superoxide dismutase, Fe-Mn family [EC:1.15.1.1] | *sodB* | KEGG |
| GC_00001730 | FAD-dependent pyridine nucleotide-disulphide oxidoreductase | *-* | PFAM |
| GC_00001831 | Hypothetical protein |  | - |
| GC_00001854 | Large subunit ribosomal protein L31 | *rpmE* | KEGG |
| GC_00001981 | HSP20 family protein | *hspA* | KEGG |
| GC_00002043 | Cytochrome c oxidase subunit III [EC:7.1.1.9] | *coxC* | KEGG |
| GC_00002064 | CBS domain | *-* | eggNOG |
| GC_00002175 | Cytochrome c oxidase subunit II [EC:7.1.1.9] | *coxB* | KEGG |
| GC_00002201 | Hypothetical protein | *-* | - |
| GC_00002227 | Part of a sulfur-relay system | *tusE* | eggNOG |
| GC_00002429 | DsrE/DsrF/DrsH-like family | *-* | eggNOG |
| GC_00002461 | Rrf2 family transcriptional regulator, iron-sulfur cluster assembly transcription factor | *iscR* | KEGG |
| GC_00002523 | Translation initiation factor IF-3 | *infC* | KEGG |
| GC_00002666 | Lanthanide-dependent methanol dehydrogenase [EC:1.1.2.10] | *xoxF* | KEGG |
| GC_00002684 | Belongs to the small heat shock protein (HSP20) family | *-* | eggNOG |
| GC_00002714 | RNA recognition motif | *-* | PFAM |
| GC_00002731 | Cytochrome subunit of sulfide dehydrogenase | *cc4* | KEGG |
| GC_00002788 | Host factor-I protein | *hfq* | KEGG |
| GC_00002946 | Integral membrane protein (DUF2244) | *-* | eggNOG |
| GC_00003028 | Dissimilatory sulfite reductase related protein | *dsrC* | KEGG |
| GC_00003200 | Hypothetical protein | *-* | - |
| GC_00003451 | Ala-tRNA(Pro) deacylase [EC:3.1.1.-] | *prdX* | KEGG |
| GC_00003489 | Hypothetical protein | *-* | - |

### Genes exclusively transcribed by *Ca.* T. boucheti indicate potential differences in symbiont lifestyle within the host environment

Within the orthogroups that were exclusively and consistently transcribed by *Ca.* T. boucheti were several genes that encoded type IV pili proteins (minor pilin, assembly proteins and secretin) (Table III). Type IV pili are known molecular mechanisms involved in animal-microbe symbioses: they are associated with a number of processes, including adhesion to host cells, biofilm formation, twitching motility, and protein secretion [(7)](https://paperpile.com/c/Mmzmxv/Ddj0i). Closely related lucinid symbionts co-exist within the same host by occupying distinct bacteriocytes in the gills [(6)](https://paperpile.com/c/Mmzmxv/9ZxU5) and this compartmentalization could prevent competitive interactions between the symbionts for limited resources. However, further studies are required to understand how these transcriptional differences among distinct co-existing symbiont clades affect host colonization mechanisms or host-microbe crosstalk and whether this allows the host to distinguish different members of the community.

**Table III.** Orthogroups exclusively and constitutively expressed by *Ca.* Thiodiazotropha boucheti.

| orthogroup ID | Intersection | Kofam | *Ca.* T.  boucheti  UPW  Median TPM | *Ca.* T. endolucinida  UPW  Median TPM | *Ca.* T. boucheti  nUPW  Median TPM | *Ca.* T. endolucinida  nUPW  Median TPM |
| --- | --- | --- | --- | --- | --- | --- |
| GC_00000074 | boucheti_UPW - boucheti_nUPW | type IV fimbrial biogenesis protein FimT | 242.75 | 7.805 | 299.91 | 0 |
| GC_00000070 | boucheti_UPW - boucheti_nUPW | type IV pilus assembly protein PilE | 1871.87 | 0 | 2380.55 | 0 |
| GC_00000108 | boucheti_UPW - boucheti_nUPW | type IV pilus assembly protein PilN | 125.34 | 1.47 | 173.22 | 0.89 |
| GC_00002001 | boucheti_UPW - boucheti_nUPW | type IV pilus assembly protein PilO | 61.42 | 0.06 | 56.13 | 0 |
| GC_00000109 | boucheti_UPW - boucheti_nUPW | type IV pilus assembly protein PilQ | 268.84 | 0.515 | 283.21 | 1.65 |
| GC_00003589 | boucheti_UPW - boucheti_nUPW | type IV pilus assembly protein PilV | 139.12 | 0 | 198.12 | 0 |
| GC_00000106 | boucheti_UPW - boucheti_nUPW | type IV pilus assembly protein PilW | 14.02 | 3.71 | 10.95 | 0 |
| GC_00001974 | boucheti_UPW - boucheti_nUPW | type IV pilus assembly protein PilW | 106.27 | 0 | 180.65 | 0 |
| GC_00000116 | boucheti_UPW - boucheti_nUPW | type IV pilus assembly protein PilX | 10.41 | 0 | 20.05 | 0 |
| GC_00003599 | boucheti_UPW - boucheti_nUPW | type IV pilus assembly protein PilX | 205 | 0 | 197.65 | 0 |

### Exclusive clade functions are not exploited in response to upwelling

To identify any potential clade-specific genomic adaptation to the seasonal upwelling, we characterized the metabolic functions exclusive to each symbiont clade and found 23 KOfam functions enriched in the MAGs of *Ca.* T. boucheti and 21 in the MAGs of *Ca.* T. endolucinida TEP (Table IV). The genes conferring clade-specific metabolic functions showed no significant changes across seasons (Table IV), suggesting that they are not involved in the response to upwelling-driven environmental changes. These clade-specific differences in genome content could be more advantageous to the symbionts during their free-living phase when they might contribute to niche differentiation. Furthermore, associating with functionally redundant partners may provide resilience to the lucinid symbiosis against fluctuations in the availability of suitable symbiont candidates in free-living populations.

**Table IV.** Differential expression analysis results of genes associated to enriched funtions in *Ca.* T. boucheti and endolucinida.

| Gene Definition | Clade enriched | Base  Mean | log2Fold  Change | lfcSE | pvalue | padj |
| --- | --- | --- | --- | --- | --- | --- |
| 1417 3 hydroxybutyrate dehydrogenase K00019 | *Ca.*  T boucheti | 5.829358469 | 0.035572342 | 0.32181407 | 0.921154851 | 1 |
| 1418 3 hydroxybutyrate dehydrogenase K00019 | *Ca.*  T boucheti | 9.280855597 | 0.035747313 | 0.286593562 | NA | NA |
| 3506 toxin HigB 1 K07334 | *Ca.*  T boucheti | 2.420774074 | 0.133119369 | 0.437232793 | 0.331471899 | 1 |
| 3121 two component system sensor histidine kinase K20974 | *Ca.*  T boucheti | 8.75488042 | -0.12569391 | 0.250038398 | 0.64097783 | 1 |
| 4058 osmotically inducible lipoprotein OsmB | *Ca.*  T boucheti | 3.714506989 | -0.270928478 | 0.486038075 | 0.136848041 | 1 |
| 2023 MerR family transcriptional regulator light induced transcriptional regulator K22491 | *Ca.*  T boucheti | 90.59297297 | 0.177811859 | 0.344451612 | 0.320025546 | 1 |
| 3537 phosphoribosylglycinamide formyltransferase 2 K08289 | *Ca.*  T boucheti | 41.26966463 | 0.19270381 | 0.243989351 | 0.463960096 | 1 |
| 3529 arsenite transporter K03325 | *Ca.*  T boucheti | 10.2061611 | -0.20236974 | 0.353587113 | 0.269524088 | 1 |
| 2019 glutathione peroxidase K00432 | *Ca.*  T boucheti | 411.1394567 | -0.037707948 | 0.321608987 | 0.906635941 | 1 |
| 3866 two component system sensor histidine kinase K20974 | *Ca.*  T boucheti | 8.150481197 | -0.094800194 | 0.286866656 | 0.662070538 | 1 |
| 430 S adenosylmethionine decarboxylase K01611 | *Ca.*  T boucheti | 20.19709233 | -0.242913316 | 0.32313329 | 0.239953365 | 1 |
| 931 LysR family transcriptional regulator transcriptional activator for bauABCD operon K21699 | *Ca.*  T boucheti | 10.12841431 | -0.040922041 | 0.21995565 | 1 | 1 |
| 930 beta alanine pyruvate transaminase K00822 | *Ca.*  T boucheti | 4.951562686 | 0.086510854 | 0.301930584 | 0.66512558 | 1 |
| 1415 uncharacterized oxidoreductase K14189 | *Ca.*  T boucheti | 5.452301259 | -0.010725602 | 0.318747145 | NA | NA |
| 2513 trypsin K01312 | *Ca.*  T boucheti | 14.11178202 | 0.108110501 | 0.306877719 | 0.564369272 | 1 |
| 929 malonate semialdehyde dehydrogenase acetylating methylmalonate semialdehyde dehydrogenase K00140 | *Ca.*  T boucheti | 2.658409761 | 0.224108946 | 0.50476484 | 0.165888972 | 1 |
| 918 transcriptional regulator of aroF aroG tyrA and aromatic amino acid transport K03721 | *Ca.*  T boucheti | 10.27071436 | 0.101609335 | 0.280681167 | 0.648331233 | 1 |
| 919 D amino acid dehydrogenase K00285 | *Ca.*  T boucheti | 6.180791934 | -0.289166127 | 0.442453732 | 0.13545145 | 1 |
| 1918 toxin HigB 1 K07334 | *Ca.*  T boucheti | 24.26885788 | 0.025254159 | 0.211860496 | 1 | 1 |
| 1423 acetoin 2 6 dichlorophenolindophenol oxidoreductase subunit beta K21417 | *Ca.*  T boucheti | 18.98550993 | -0.237614613 | 0.420634131 | 0.185518553 | 1 |
| 4239 3alpha or 20beta hydroxysteroid dehydrogenase K00038 | *Ca.*  T boucheti | 0.186737701 | -0.012682318 | 0.450353722 | 0.942150262 | 1 |
| 3666 putative salt induced outer membrane protein K07283 | *Ca.*  T boucheti | 15.69505644 | -0.012426843 | 0.238237191 | 1 | 1 |
| 428 ornithine decarboxylase K01581 | *Ca.*  T boucheti | 10.75142351 | -0.570798583 | 0.46497958 | 0.031467225 | 0.831740053 |
| 605 DNA transformation protein and related proteins K07343 | *Ca.*  T boucheti | 0.299626808 | 0.071086999 | 0.41901977 | 0.561480215 | 1 |
| 1426 aldehyde dehydrogenase NAD K00128 | *Ca.*  T boucheti | 7.375296806 | -0.539985154 | 0.593012948 | NA | NA |
| 1427 guanidinopropionase K18459 | *Ca.*  T boucheti | 3.371552396 | -0.280804262 | 0.488284016 | 0.129854212 | 1 |
| 287 putative acyl CoA dehydrogenase | *Ca.*  T endolucinidaTEP | 6.053932029 | -0.177642649 | 0.359350421 | 0.44269619 | 1 |
| 288 putative acyl CoA dehydrogenase | *Ca.*  T endolucinidaTEP | 2.099343769 | -0.056689617 | 0.353227106 | 0.888680398 | 1 |
| 3094 D lactate dehydrogenase | *Ca.*  T endolucinidaTEP | 2.47764928 | -0.027009419 | 0.395050687 | 0.958724899 | 1 |
| 3096 D lactate dehydrogenase | *Ca.*  T endolucinidaTEP | 1.85946861 | -0.483392948 | 0.678059515 | 0.027823622 | 0.492928135 |
| 390 undecaprenyl phosphate alpha L ara4FN deformylase K13014 | *Ca.*  T endolucinidaTEP | 9.2551337 | -0.146528036 | 0.322488735 | 0.601496821 | 1 |
| 2991 SARP family transcriptional regulator regulator of embCAB operon K22894 | *Ca.*  T endolucinidaTEP | 118.2576637 | -0.097239544 | 0.267441707 | 0.894104149 | 1 |
| 385 sarcosine dimethylglycine N methyltransferase K18897 | *Ca.*  T endolucinidaTEP | 14.86852938 | 0.211223512 | 0.31157473 | 0.455841625 | 1 |
| 1592 protein PsiE K13256 | *Ca.*  T endolucinidaTEP | 3.895314386 | -0.189992969 | 0.37397729 | 0.387990721 | 1 |
| 66 hemolysin III K11068 | *Ca.*  T endolucinidaTEP | 59.17113197 | 0.013419658 | 0.183792689 | 1 | 1 |
| 432 TetR AcrR family transcriptional regulator copper responsive repressor K22041 | *Ca.*  T endolucinidaTEP | 81.79307322 | 0.038420974 | 0.310345115 | 1 | 1 |
| 286 protocatechuate 3 4 dioxygenase beta subunit K00449 | *Ca.*  T endolucinidaTEP | 69.06602392 | -0.114318981 | 0.333695914 | 0.684396287 | 1 |
| 391 ribosomal protein S5 alanine N acetyltransferase K03790 | *Ca.*  T endolucinidaTEP | 2.429419946 | -0.026862404 | 0.373171163 | 1 | 1 |
| 2679 UDP N acetyl 3 dehydro alpha D glucosamine 3 aminotranferase K20084 | *Ca.*  T endolucinidaTEP | 26.24394827 | 0.079023104 | 0.226657461 | 1 | 1 |
| 3892 LysR family transcriptional regulator transcriptional activator of nhaA K03717 | *Ca.*  T endolucinidaTEP | 14.96003907 | -0.407748559 | 0.433174744 | 0.10492445 | 0.82176617 |
| 200 aminoglycoside 6 N acetyltransferase I K18816 | *Ca.*  T endolucinidaTEP | 2.431556134 | 0.063696416 | 0.380638047 | 0.781471873 | 1 |
| 3598 fluoroacetyl CoA thioesterase K18700 | *Ca.*  T endolucinidaTEP | 0.838819402 | -0.120585648 | 0.451368739 | 0.353389429 | 1 |
| 3843 AraC family transcriptional regulator regulatory protein of adaptative response methylated DNA protein cysteine methyltransferase K10778 | *Ca.*  T endolucinidaTEP | 44.80663063 | -0.170815245 | 0.291089547 | 0.590100584 | 1 |
| 2034 NADH dependent peroxiredoxin subunit F K03387 | *Ca.*  T endolucinidaTEP | 48.61439582 | 0.020363055 | 0.246999032 | 1 | 1 |
| 2445 alginate production protein K16081 | *Ca.*  T endolucinidaTEP | 7.203485484 | -0.069953824 | 0.316373644 | 0.911709939 | 1 |
| 3880 cardiolipin synthase A B K06131 | *Ca.*  T endolucinidaTEP | 23.83663304 | -0.3087363 | 0.352699831 | 0.243632896 | 1 |
| 2335 transcriptional regulator HilA main transcriptional regulator of SPI1 K22486 | *Ca.*  T endolucinidaTEP | 32.58652241 | 0.122556921 | 0.2625617 | 0.800768348 | 1 |
| 3891 universal stress protein E K14055 | *Ca.*  T endolucinidaTEP | 11.76333585 | -0.281081002 | 0.361282681 | 0.267453786 | 1 |
| 3398 tetratricopeptide repeat protein 38 K24942 | *Ca.*  T endolucinidaTEP | 17.22346861 | -0.531597478 | 0.463030732 | 0.050900248 | 0.635760899 |

## Supplementary references

1. [Seeman T. 2013. barrnap 0.9 : rapid ribosomal RNA prediction.](http://paperpile.com/b/Mmzmxv/bPcY)

2. [Shaiber A, Willis AD, Delmont TO, Roux S, Chen L-X, Schmid AC, Yousef M, Watson AR, Lolans K, Esen ÖC, Lee STM, Downey N, Morrison HG, Dewhirst FE, Mark Welch JL, Eren AM. 2020. Functional and genetic markers of niche partitioning among enigmatic members of the human oral microbiome. Genome Biol 21:292.](http://paperpile.com/b/Mmzmxv/0Kls6)

3. [Berndt V, Beckstette M, Volk M, Dersch P, Brönstrup M. 2019. Metabolome and transcriptome-wide effects of the carbon storage regulator A in enteropathogenic Escherichia coli. Sci Rep 9:138.](http://paperpile.com/b/Mmzmxv/7t4KI)

4. [Henderson B. 2008. Cell stress proteins as modulators of bacteria--host interactions. Novartis Found Symp 291:141–54; discussion 154–9, 221–4.](http://paperpile.com/b/Mmzmxv/CUJdj)

5. [Lim SJ, Alexander L, Engel AS, Paterson AT, Anderson LC, Campbell BJ. 2019. Extensive thioautotrophic gill endosymbiont diversity within a single *Ctena orbiculata* (Bivalvia: Lucinidae) Population and implications for defining host-symbiont specificity and species recognition. mSystems 4.](http://paperpile.com/b/Mmzmxv/tZ9q9)

6. [Osvatic JT, Wilkins LGE, Leibrecht L, Leray M, Zauner S, Polzin J, Camacho Y, Gros O, van Gils JA, Eisen JA, Petersen JM, Yuen B. 2021. Global biogeography of chemosynthetic symbionts reveals both localized and globally distributed symbiont groups. Proc Natl Acad Sci U S A 118.](http://paperpile.com/b/Mmzmxv/9ZxU5)

7. [Ganesan R, Wierz JC, Kaltenpoth M, Flórez LV. 2022. How it all begins: Bacterial factors mediating the colonization of invertebrate hosts by beneficial symbionts. Microbiol Mol Biol Rev 86:e0012621.](http://paperpile.com/b/Mmzmxv/Ddj0i)
